# Approaches to Reducing Risk of COVID-19 Infections in Prisons and Immigration Detention Centers: A Commentary

**DOI:** 10.1177/0734016820957707

**Published:** 2020-09-18

**Authors:** Kate Kelly, Nai Soto, Nadi Damond Wisseh, Shaina A. Clerget

**Affiliations:** 1 School of Social Work, College of Public Health, 6558 Temple University, Philadelphia, PA, USA

**Keywords:** prison, detention, immigration, public health, coronavirus

## Abstract

Although often left out of public health efforts and policy decisions, prisons, jails, and detention centers are integral to community health. With an average of 650,000 citizens returning home from prison each year in the United States, and thousands of correctional staff members returning home every night, there are millions of touchpoints between outside communities and carceral settings. For this reason, carceral communities should be central to planning and policy making in response to the spread of the COVID-19 illness. As social workers and clinicians, we are urgently concerned that efforts to prevent COVID-19 infections in prisons are underdeveloped and inadequate in the face of a fast-spreading virus. In this commentary, we outline a set of public health, policy, and clinical recommendations based upon the existing literature to mitigate various risks to the well-being of carceral communities.

In the United States, there are 2.3 million people held in prisons, jails, juvenile facilities, and immigration detention centers, overseen by a myriad of jurisdictions and agencies (Sawyer & Wagner, 2020). The outbreak of coronavirus illness (COVID-19) has made these Americans uniquely vulnerable, largely due to physical and bureaucratic conditions of U.S. carceral systems. One of the foremost recommendations by the Center for Disease Control (CDC) to stop and slow the spread of COVID-19 is to practice social distancing and maintain a prescribed distance. However, incarcerated residents in many U.S. correctional settings face overcrowded living conditions that prevent the possibility of physical distancing. A spatial examination of cruise ships and nursing homes (settings that have been observed to promote rapid viral spread) found that prison cells are comparable sizes or smaller, often shared, and do not accommodate the 6 ft. of personal space recommended by the CDC and World Health Organization (Kajstura & Landon, 2020). Although the current overall occupancy rate of U.S. prisons is just over 103% occupancy, overcrowding varies widely by facility and state, with some prisons reporting occupancy rates up to 319% (U.S. Department of Justice, 2019). Unable to safely separate from each other, prisoners are more likely to be denied access to personal protective equipment (PPE) and disinfectant—despite national reports of prison labor being used to produce these items (Alexander, 2020; Michaels, 2020).

Emerging data show that COVID-19 disproportionately affects those who are over 65 years old, are immunosuppressed, or suffer from common chronic illnesses (Mueller et al., 2020). This puts occupants of carceral settings at increased risk on multiple levels. Due in part to past legal precedent mandating longer sentences for narcotics offenses, U.S. prisoners are increasingly aging; people over 50 now make up 16% of the state and federal prison populations, and the demographic is expected to continue growing (Skarupski et al., 2018). Furthermore, incarcerated people experience an increased comorbidity burden; more than half of U.S. federal prisoners report suffering from underlying conditions such as cancer, hypertension, diabetes, HIV, asthma, and/or heart disease, conditions which may increase the risk of severe illness or death if COVID-19 infection occurs (Maruschak et al., 2015; Skarupski et al., 2018).

While immigration detention facilities face the same risk factors as prisons, unique conditions and challenges further threaten the health of 43,000 people in Immigration & Customs Enforcement (ICE) custody nationally. In some regions, individuals arrested by ICE are held in local jails and prisons, while other regions utilize designated, immigration-specific detention centers. These facilities frequently lack medical infrastructure necessary for treating severe COVID-19 cases, and a third of detainees are held in communities with one or zero hospitals that have intensive care units. This suggests that outbreaks at detention facilities located in rural areas could contribute to wider local infection that might overwhelm a small community’s medical services (Cooke, 2020). Furthermore, in a rapidly evolving pandemic, translation services in detention can quickly become interrupted. This diminishes detainees’ access to information about their own health and personal risk, effectively turning a language barrier into a comorbidity factor.

## COVID-19 Infections in Prisons and Immigration Detention Centers: A Call for Evidence-Based Recommendations

The issue of how to protect prisoners during a pandemic is an international quandary and has resulted in a patchwork of policies varying by country, state, and municipality. Italy halted personal prison visitation with hopes of preventing illness, an act that resulted in riots and escapes across the country (Mahbubani, 2020). Faced with the pressure of mounting COVID-19 infections, Iran moved to release 85,000 prisoners nationally, prompting expressions of concern from United Nations officials (Nebehay, 2020). Absent federal recommendations, guidance, and resources to protect incarcerated individuals, state and municipal justice systems adapted independently and were unable to prevent infections from occurring in their facilities. New York City’s infamous Rikers Island jail, long a symbol of the hazards of mass incarceration, saw rapid viral spread nearly 6 times the rate of the larger city, as well as multiple early deaths among inmates and correctional officers (New York City Legal Aid Society, 2020). As of August 4, 2020, of the largest reported clusters in the country, with more than 1,000 confirmed COVID-19 cases in each setting, 13 of the 15 were correctional facilities. Despite evident similarities in the risk factors for carceral settings, recommending and implementing protective measures for prisoners is made enormously complex by differences across local, state, and federal jurisdictions, as well as disparate conditions in facilities that are privately or publicly managed. Without further prioritization and intervention, carceral settings across the United States may continue to see elevated transmission and morbidity rates.

There are multiple levels at which swift, decisive, and collaborative actions could reduce the risk of infection for incarcerated individuals and immigrant detainees. At the macro level, the federal government should protect public health by including carceral settings in comprehensive pandemic response policy, offering guidance to states, designating funding to criminal justice agencies, and prioritizing imprisoned peoples’ access to testing, vaccines, and treatment. At the mezzo level, municipalities and state-level agencies should enact protective policy measures, including delayed and reduced arrests, expanding eligibility guidelines for release, permanently decreasing incarcerated populations, and safely and ethically utilizing electronic monitoring and remote supervision. At the micro level, clinicians and advocates should assist in the equitable implementation of these policies, coordinate translation services for speakers of languages other than English, and help facilitate ethical releases through involvement in reentry planning.

## Macro-Level Public Health Efforts Grounded in Collaborative Approaches

The justice system is reliant on legal precedent and has struggled to adapt to unprecedented circumstances created by the COVID-19 pandemic. Lacking experience with placing criminal justice actions in a public health framework, municipalities have had to quickly revise and implement new policies without past reference. This has resulted in delayed, intermittent efforts that have exacerbated underlying risks for incarcerated residents and correctional staff members. Facing the likelihood of future widespread outbreaks of COVID-19 infection, courts and prisons must establish this protective precedent now to be implemented without hesitation in the future. Prosecutors, public defenders, community members, social workers, judges, court staff, police, and prison commissioners must coordinate and collaborate in their efforts to respond to emergent pandemics. Furthermore, successful legal adjustments should be shared nationally to assist other jurisdictions in navigating an outbreak.

Accepting that carceral settings are integral to larger public health efforts, federal and state governing entities must prioritize their access to PPE, as well as disease detection and treatment. Already, the spread of COVID-19 has revealed an inequality in PPE and testing availability, raising questions about who would have access to any future vaccines that may be developed (Twohey et al., 2020). Comprehensive aid packages related to COVID-19 must include designated funding to criminal justice settings, ensuring adequate supplies of PPE, tests, and treatment, in order to mitigate access inequalities and protect inmates and staff. Federal- and state-level guidance should be given on how carceral settings can coordinate with local medical services and participate in larger public health education efforts.

Finally, federal officials and policy actors should acknowledge that current conditions in correctional settings represent a significant barrier to health promotion and disease prevention, not only in the current context of COVID-19 but also in the case of any future novel virus or pandemic illness. Falling crime rates across the United States during the pandemic, including in cities that made significant prisoner releases at the onset of the U.S. COVID-19 outbreak, suggest that physical incarceration of justice-involved individuals may not be integral to public safety during a pandemic (Coyne, 2020). Moving forward, continual efforts should be made at every level to explore alternative policies that keep carceral populations as low as possible, given the heightened risk to human health in these settings. Specifically, we would recommend forming collaborative task forces at local, state, and federal levels. These task forces should include representation from community members, correctional officials, public defenders, police officers, health officials, and judges to explore alternatives to incarceration and orient system practices toward public health.

## Mezzo-Level Policy Actions to Reduce Health Risk

Most incarcerated people in the United States are being held in state and municipal facilities, representing an important level at which to intervene and enact collaborative, protective practices. In line with the decarceration priority recommended above, in order to diminish local prison populations, law enforcement agencies must agree to reduce inputs (arrests) and judges and attorneys must agree to increase outputs (releases) simultaneously. In cities like Philadelphia, agencies seemed to gravitate toward this common goal and managed a small reduction to the jail population, but the practices lacked explicit commitment. All criminal justice stakeholders, from the police to prison commissioners to public defenders to the host communities, should be united and coordinated in the common goal of creating safe conditions for incarcerated people.

There are numerous status quo carceral practices that must change in the new context of COVID-19, from routine transfers and deportations to standard probationary procedure. Once a viral outbreak is underway and local community spread has been identified, adding and transferring new individuals to carceral settings introduces significantly higher health risks to inmates and staff. During the flu pandemic of 1918, San Quentin State Prison experienced three separate waves of infection, all thought to be introduced by new arrivals to the prison (Stanley, 1919). This speaks to the need to halt routine transfers within detention systems as well as to delay and diminish the number of new people being placed in custody. Furthermore, correctional staffing practices represent a significant risk of COVID-19 to be introduced to a facility or transmitted from within a prison to their families and communities. Reducing the occurrence of procedures that require increased staff-resident contact (such as transfers and intake) and providing adequate safety training and PPE to correctional officers are essential practices.

Although some U.S. municipalities acted quickly to reduce inputs to their local prison systems by delaying arrests for certain nonviolent crimes, ICE has proceeded with immigration raids, detentions, and controversial deportations. In one instance, ICE deported COVID-exposed individuals to Haiti, a country ill-equipped to manage an outbreak of coronavirus illnesses (Del Valle & Herrera, 2020). Immigration violations are classified as civil, noncriminal offenses, and undocumented immigrants represent a low risk to public safety, suggesting these detentions are nonessential in the context of a pandemic (Orrenius & Zavodny, 2019). To mitigate risk to people in their detention facilities, ICE should halt deportations and release detainees so that they can safely practice physical distancing in alternative housing while awaiting resolution of their immigration cases.

Probation and parole services is another avenue to implement new practices that further reduce interpersonal interaction and prevent COVID-19 transmission. The remote supervision model, including phone check-ins and electronic monitoring, represents an interesting opportunity to protect public health. This prevents thousands of instances of individuals from leaving their homes or residential programs, utilizing public transportation, and entering buildings with probation employees, activities that can increase the spread of COVID-19. As U.S. medical services migrate their practice to telemedicine (digital, remote appointments in order to evaluate patients without risk of COVID-19 exposure), probationary and parole agencies are considering a similar shift. However, overreliance on electronic monitoring could result in a replication of harmful carceral conditions outside of prison, expanding opportunities for punitive control into homes and neighborhoods (Schenwar & Law, 2020). While a shift to remote probation would be beneficial to preventing the spread of COVID-19, it must be undertaken carefully to mitigate unethical effects. Noncorrectional social workers and case managers who work with people in reentry must also be aware of ethical implications of remote service as well as disparities in technology access.

## Micro-Level Clinical Advocacy Based in Trauma-Informed Practice

Within larger criminal justice systems, social workers, clinicians, attorneys, and other advocates have a valuable role to play in protecting the health of carceral communities through targeted, trauma-informed interventions that promote health. “Trauma-informed” clinical practice entails providing care under the assumption that individuals may have a history of trauma and with the goal of preventing retraumatization (Knight, 2015). A widespread global pandemic has potential negative mental health implications for all members of society, but incarcerated individuals may be acutely affected, given that rates of personal trauma history among incarcerated people are high (Jäggi et al., 2016; Wolff & Shi, 2012). As discussed by Hewson et al. (2020), prisoners may currently face increased anxiety and uncertainty due to a lack of visitation, limited access to reliable information, and indefinitely extended court dates. It follows that incarcerated people may need increased clinical support and access to mental health treatment as a result of COVID-19 outbreaks in their facilities and communities.

To help mitigate health risks and anxieties, carceral clinicians should play a forefront role in distributing PPE to incarcerated people and should lead efforts to provide education about the virus and prevention measures. Prison social workers and medical staff also have a professional imperative to advocate for adequate treatment of COVID-19 illness in prisoners to protect the lives of infected individuals as well as the wider community. To overcome language barrier risks to the health of people in immigration detention centers, clinicians in these settings should ensure that information is provided in individuals’ native languages to ensure comprehension and adherence to prevention efforts. One model that has already emerged in this area is the Crisis Translators Network (n.d.), an association of volunteer translators who have responded to the pandemic by coordinating trauma-informed language services in hospitals, detention centers, and community settings.

Clinical intervention would benefit not only the health of incarcerated individuals but also the health of those in reentry. Prior to the emergence of COVID-19, individuals released from prison faced heightened barriers in obtaining housing, employment, medical care, and mental health treatment, challenges which may be further exacerbated by pandemic conditions (Semenza & Link, 2019). Individuals released from Rikers Island in the midst of New York’s rapidly rising COVID-19 infections reported receiving no instructions or information related to disease prevention upon their release (Al-Hlou et al., 2020). The protective benefits of releasing people from incarceration are nullified if individuals lack housing, food, or essential health information. To ensure releases are ethical and beneficial to public health, social workers and advocates should be closely involved in reentry planning.

## Conclusions

It is clear that carceral communities can no longer be excluded from comprehensive planning to prevent COVID-19 outbreaks. Structural limitations, comorbidity factors, and resource disparities create heightened risk within correctional facilities and the wider communities with which they interact. Furthermore, protecting incarcerated individuals during a pandemic is not only a matter of public health, it is a matter of civil rights and racial justice. Mass incarceration has long been acknowledged to have a disproportionate effect on communities of color, and emergent data suggest that across the United States, Black, Latinx, and Native individuals are being infected with COVID-19 and dying from the disease at higher rates than White people. To mitigate these injustices and protect wider public health, justice officials should work to permanently reduce carceral populations, provide protective resources, and enact the clinical recommendations outlined above.
